# The phenotype of a knockout mouse identifies flavin-containing monooxygenase 5 (FMO5) as a regulator of metabolic ageing

**DOI:** 10.1016/j.bcp.2015.05.013

**Published:** 2015-08-01

**Authors:** Sandra G. Gonzalez Malagon, Anna N. Melidoni, Diana Hernandez, Bilal A. Omar, Lyndsey Houseman, Sunil Veeravalli, Flora Scott, Dorsa Varshavi, Jeremy Everett, Yugo Tsuchiya, John F. Timms, Ian R. Phillips, Elizabeth A. Shephard

**Affiliations:** a Institute of Structural and Molecular Biology, University College London, London WC1E 6BT, UK; b School of Biological and Chemical Sciences, Queen Mary University of London, London E1 4NS, UK; c Medway Metabonomics Research Group, University of Greenwich, Chatham Maritime, Kent ME4 4TB, UK; d Women's Cancer, Institute for Women's Health, University College London, London WC1E 6BT, UK

**Keywords:** Body weight, Cholesterol, Glucose, Malic enzyme 1, White adipose tissue

## Abstract

We report the production and metabolic phenotype of a mouse line in which the *Fmo5* gene is disrupted. In comparison with wild-type (WT) mice, *Fmo5*^*−/−*^ mice exhibit a lean phenotype, which is age-related, becoming apparent after 20 weeks of age. Despite greater food intake, *Fmo5*^*−/−*^ mice weigh less, store less fat in white adipose tissue (WAT), have lower plasma glucose and cholesterol concentrations and enhanced whole-body energy expenditure, due mostly to increased resting energy expenditure, with no increase in physical activity. An increase in respiratory exchange ratio during the dark phase, the period in which the mice are active, indicates a switch from fat to carbohydrate oxidation. In comparison with WT mice, the rate of fatty acid oxidation in *Fmo5*^*−/−*^ mice is higher in WAT, which would contribute to depletion of lipid stores in this tissue, and lower in skeletal muscle. Five proteins were down regulated in the liver of *Fmo5*^*−/−*^ mice: aldolase B, ketohexokinase and cytosolic glycerol 3-phosphate dehydrogenase (GPD1) are involved in glucose or fructose metabolism and GPD1 also in production of glycerol 3-phosphate, a precursor of triglyceride biosynthesis; HMG-CoA synthase 1 is involved in cholesterol biosynthesis; and malic enzyme 1 catalyzes the oxidative decarboxylation of malate to pyruvate, in the process producing NADPH for use in lipid and cholesterol biosynthesis. Down regulation of these proteins provides a potential explanation for the reduced fat deposits and lower plasma cholesterol characteristic of *Fmo5*^*−/−*^ mice. Our results indicate that disruption of the *Fmo5* gene slows metabolic ageing via pleiotropic effects.

## Graphical abstract

**Figure:**
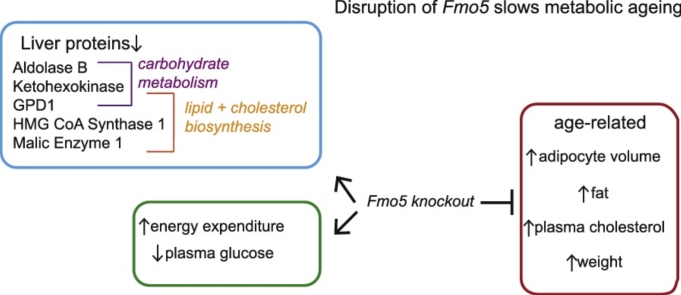

## 1 Introduction

Flavin-containing monooxygenases (FMOs) (EC 1.14.13.8) of eukaryotes are located in the membranes of the endoplasmic reticulum. Humans possess five functional *FMO* genes [1,2], four of which, *FMO1*, *2*, *3* and *4*, are clustered on chromosome 1, in the region 1q24.3, whereas *FMO5* is located at 1q21.1 [1]. In mouse, the genes encoding FMOs 1, 2, 3 and 4 are clustered on Chromosome 1 and *Fmo5* is on Chromosome 3 [1].

FMOs 1, 2 and 3 catalyze the NADPH-dependent oxygenation of a wide array of foreign chemicals, including drugs, environmental pollutants and dietary-derived compounds [3]. The *FMO1* gene displays little genetic variation, with only a few coding-region single-nucleotide polymorphisms (SNPs), each of which is present at low frequency [2,4] and does not affect catalytic activity [5]. The majority of humans, however, are homozygous for a nonsense mutation of *FMO2*, which results in the expression of a non-functional protein [6,7], and mutations in *FMO3* cause the disorder trimethylaminuria [8], which is characterized by an unpleasant body odour, due to defective *N*-oxygenation of dietary-derived trimethylamine [9]. FMO4 has not been detected in vivo and little is known of its substrates or activity.

Although FMO5 is highly expressed in the liver of mice [10] and humans [11], little is known of the role of this protein. FMO5 displays few characteristics of an enzyme involved in the detoxification of small foreign chemicals, with little or no activity towards classic FMO substrates, such as methimazole [12], trimethylamine [13] and benzydamine [14]. Knowledge of its substrates is limited [2,15,16]: it catalyzes the *N*-oxygenation of short-chain aliphatic primary amines such as *N*-octylamine [12] and the *S*-oxygenation of *S*-methyl-esonarimod, an active metabolite of the anti-rheumatic esonarimod [15,17]. The ability of FMO5 to catalyze a Baeyer–Villiger oxidation reaction was revealed in a study of an anti-cancer therapeutic [18].

Interindividual variation in the amount of FMO5 in human liver has been reported [19]. This may be due to physiological effects or to exposure to foreign chemicals, as, unlike other FMOs, FMO5 is inducible by a number of chemicals, e.g., expression is increased in a breast cancer cell line by the synthetic progestin R5020 [20], in human hepatocytes by rifampin [21] and in HepG2 cells by hyperforin [22].

Recently, through the use of a knockout (KO) mouse line, we have shown that FMO1 plays an important role not only in drug metabolism in vivo [23,24], but also in endogenous metabolism, acting as a regulator of energy homeostasis [25]. A physiological role for FMO5 is also suspected, due to the small number of xenobiotic substrates for the enzyme and the paucity of coding-region SNPs in the *FMO5* gene. To investigate the role of FMO5 we produced a KO mouse line in which the *Fmo5* gene has been disrupted. Here, we report the metabolic phenotype of *Fmo5*^*−/−*^ mice. This indicates that FMO5 promotes metabolic ageing, via pleiotropic effects, including acting as a positive modulator of cholesterol biosynthesis, and thus identifies, for the first time, a role for this protein in endogenous metabolism.

## 2 Materials and methods

### 2.1 Gene targeting and generation of *Fmo5*^*−/−*^ mice

**Fig. 1:**
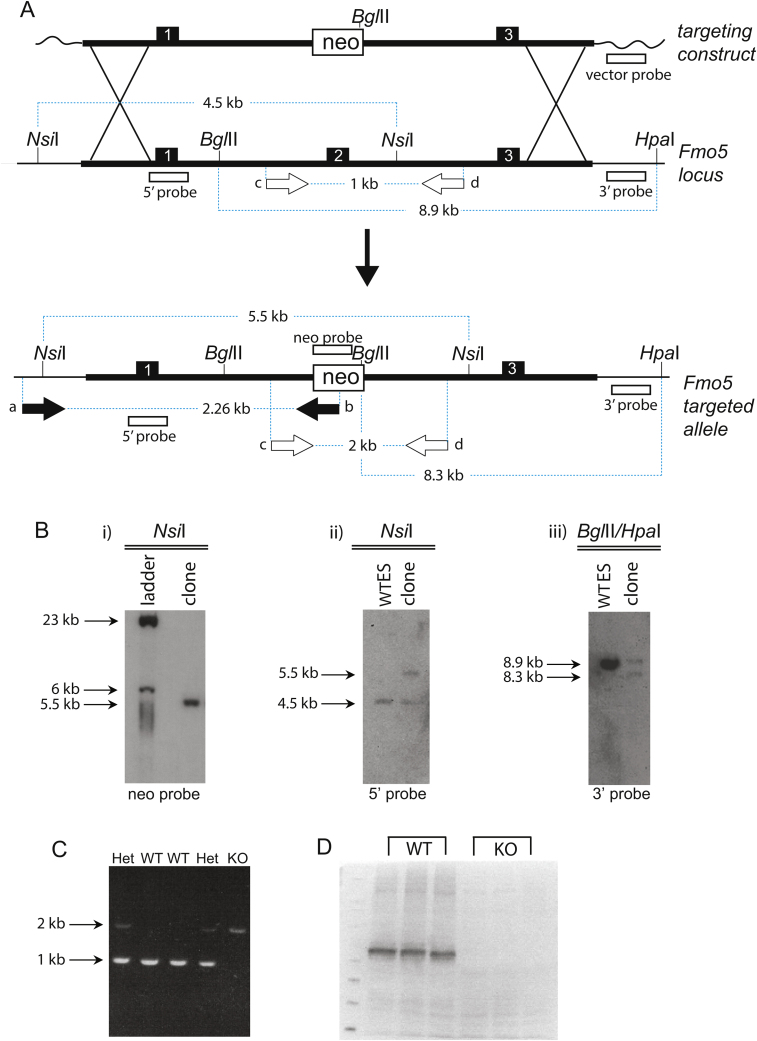
Gene targeting and generation of *Fmo5^−/−^* KO mice. (A) Recombination between homologous regions (thick black lines) of the targeting construct and the *Fmo5* locus leading to the generation of the *Fmo5* targeted allele, in which exon 2 is replaced by the *neo^r^* cassette (neo). Exons are represented by black boxes. Primers used in PCR screening of the G418^r^ ES clones are depicted with black arrows (a and b). Primers used to distinguish between the WT and targeted alleles are depicted with white arrows (c and d) (see also C). Restriction sites and probes used for Southern blot genotyping of the targeted ES clones and the mice are also shown. Not drawn to scale. (B) After PCR screening (not shown) Southern blot analysis with (i) a *neo*^r^-specific probe, (ii) a 5′-internal probe, (iii) a 3′-external probe further confirmed the correct structure of the targeted allele. Analysis using a vector-specific probe verified the absence of any random integrations in the targeted ES clones (data not shown). (C) Mice were genotyped by PCR analysis of tail DNA with primers c and d (see A). Het, heterozygous knockout (*Fmo5*^+/−^); KO, homozygous knockout (*Fmo5*^−/−^); WT, wild type. (D) Western blot of liver proteins showing lack of FMO5 expression in *Fmo5*^−/−^ mice (KO) compared with WT mice (WT).

Genomic clones containing *Fmo5* were isolated from the RPC1-21 mouse Pac genomic library (Roswell Park Cancer Institute, Buffalo, NY) by screening with a PCR product corresponding to the 3′-UTR of human *FMO5*. The identity of the clones was confirmed by hybridization with the 3′-UTR of mouse *Fmo5* and restriction map comparison with mouse genomic DNA. One of the clones (in a pPAC4 vector) was analyzed by Southern blotting with *Fmo5* exon-specific PCR products as probes. This identified the appropriate restriction fragments to be incorporated in the targeting vector and the screening strategy to be employed at the embryonic stem (ES) cell and animal level. A 2-kb *Kpn*I fragment extending upstream from a site located 150 bp upstream of the ATG translational initiation codon and a 7-kb *Apa*I fragment extending downstream from a site located 653 bp downstream of the translational initiation codon were used as the short and long arms of homology, respectively. These fragments were inserted on either side of a *neo*^*r*^ cassette within a pPN targeting vector to produce the final targeting construct (Fig. 1A). pPN was modified from pPNT (a gift of VLJ Tybulewicz, MRC NIMR, Mill Hill, London) by excision of the *HSV-1tk* cassette.

E14 ES cells (derived from the 129/Ola mouse strain) were transfected, by electroporation, with the linearized targeting construct. Recombination between the homologous regions of the targeting construct and the *Fmo5* genomic locus (Fig. 1A) would result in the replacement of exon 2, which encodes the ATG translation initiation codon and the FAD-binding domain of FMO5, by the *neo*^*r*^ cassette, rendering the allele null. After positive selection of ES cells with G418, correctly targeted clones were identified by both PCR and Southern-blot analysis (Fig. 1B). ES cells in which the *Fmo5* gene had been correctly targeted were injected into C57BL/6 mouse blastocysts [26]. Blastocysts were then implanted into the uterus of a pseudopregnant CD-1 mouse [26].

Male chimeric progeny were crossed with WT female C57BL/6 mice to test for germline transmission of the ES cell mutation. Progeny arising from germ cells derived from the injected ES cells, i.e., agouti coloured, were genotyped as described below and in Fig. 1C. Progeny that were heterozygous for the disrupted *Fmo5* gene were backcrossed with WT C57BL/6 mice for eight generations to establish a congenic KO mouse line. Offspring were genotyped, by PCR analysis of mouse tail DNA, as described previously [26], using the primer pair 2a-A1r (2a, forward primer 5′ CCTTTGCGTTTACGGAAGAAGGGTGC 3′; A1r, reverse primer 5′ TTGCCTGCATGCATGTTTATGTGC 3′), to confirm the presence of the targeted allele (∼2 kb) or the wild-type allele (∼1 kb) (Fig. 1C).

### 2.2 Animal maintenance, body weight and food intake measurements

Males and females from the congenic mouse line, which were heterozygous for the disrupted *Fmo5* gene, were mated to produce the homozygous KO mice (*Fmo5*^*−/−*^*)* used in this study. The absence of the FMO5 protein in the KO animals was confirmed by western blotting (Fig. 1D). WT C57BL/6 mice were used as controls. Mice were bred at UCL and fed a standard chow diet (Teklad Global 18% Protein Rodent Diet, Harlan Laboratories, Inc., Madison, WI). For body weight measurement, animals were housed at a maximum density of five per cage and weight was recorded up to 52 weeks of age. For food intake measurement, animals were housed at a density of four per cage and food weighed before and after each 72-h period for 10 weeks. Tissue, plasma and urine samples were collected between 10:30 a.m. and 12:00 noon. Animal procedures were carried out in accordance with the UK Animal Scientific Procedures Act and with local ethics committee approval and appropriate Home Office Licences.

### 2.3 Morphological analysis

Sections of white adipose tissue (WAT) were stained with haematoxylin and eosin. Diameters of adipocytes were measured as described previously [25]. Frozen sections (12 μm) of liver were stained with Oil Red O (Sigma–Aldrich, Poole, UK) and counterstained with haematoxylin.

### 2.4 Plasma and urine metabolites

After restriction of food for either 4 h or overnight, blood and urine were collected between 10:30 a.m. and 12:00 noon. Blood samples were taken from mouse tail tips and plasma was isolated, as described [27]. Concentrations of plasma and urine metabolites, with the exception of pyruvate, were determined at the MRC Mammalian Genomics Unit, Harwell, Oxon, UK [27]. Pyruvate was quantified by NMR spectroscopy in plasma samples diluted 2:1 with 0.9% saline in D_2_O, the latter to provide the field/frequency ‘lock’ for the NMR spectrometer. ^1^H NMR spectra were recorded on an Avance spectrometer (Bruker BioSpin GmbH, Rheinstetten, Germany) operating at 600.44 MHz at a temperature of 300 K. Two types of one-dimensional (1D) ^1^H NMR spectra were acquired. The first was a standard 1D NOESY presaturation (Bruker pulse sequence noesypr1d) spectrum using the pulse sequence (RD-90°-*t*1-90°-*t*m-90°-acquire). For each spectrum, a total of eight dummy scans and 128 transients were collected into 65,536 data points over a spectral width of 20.0173 ppm, using a relaxation delay (RD) of 2 s, and a mixing time of 100 ms. To suppress signals from macromolecules, a second set of data was acquired using the Carr–Purcell–Meiboom–Gill (CPMG) spin-echo experiment (Bruker pulse sequence cpmgpr). The pulse sequence used was RD [90°*x* − (*τ* − 180°*y* − τ)_*n*_ − acquire], with RD = 2 s, *τ*, spin-echo delay = 400 μs and *n* (the number of loops) = 100, and therefore a total spin–spin relaxation delay (2*nτ*) of 80 ms. During the relaxation delay, low-power irradiation was applied to achieve suppression of the water peak. Other parameters were as above. All NMR spectra were processed using MNova software v9.1.0 (Mestrelab, Santiago de Compostela, Spain) with zero-filling to 65,536 data points, apodisation with a line-broadening of 0.3 Hz, automated baseline correction and phasing (with manual override as required) and referencing to the alpha anomeric proton of d-glucose at 5.233 ppm. Quantification of pyruvate in the NOESY presaturation spectra was made relative to the concentration of glucose (Cg) in each sample, determined by biochemical analysis. Specifically, the area for the methyl signal for pyruvate at 2.37 ppm was divided by 3 to obtain the one-proton equivalent signal area, Apyr. This was then compared with the one-proton signal area calculated for d-glucopyranose, Ag. To obtain the latter figure, the area of the alpha anomeric proton of d-glucopyranose at 5.233 ppm was divided by the known fraction of the alpha pyranose in solution: 0.376 [28]. Pyruvate concentration was then calculated as Cg × Apyr/Ag. All signal area measurements were done by manual integration in MNova.

### 2.5 Glycogen measurements

Liver glycogen was measured by the glucose oxidase assay method as described by Huijing [29].

### 2.6 Metabolic rate measurements

30-week-old male mice were housed individually in Oxymax cages (Columbus Instruments, Columbus, OH) at 27 °C and allowed to acclimatize for 24 h. VO_2_ and VCO_2_ were measured, over a 72-h period, and heat, respiratory exchange ratio (RER) and resting energy expenditure (REE) calculated, as described previously [25].

### 2.7 Assessment of voluntary exercise

30-week-old male mice were housed individually in an activity wheel chamber system (Lafayette Instrument Co., Lafayette, IN). Activity was measured every minute during the dark phase and every hour during the light phase for a period of seven days. Data were analyzed using the Lafayette software excel office add-in and the hourly average of the interval count data was calculated to determine the total wheel revolutions per hour per mouse.

### 2.8 Fatty acid oxidation

Fatty acid oxidation was measured using [U-^14^C]palmitate (GE Life Sciences, Little Chalfont, Bucks, UK), essentially as described [30].

### 2.9 Proteomic analysis

Livers from five KO and five WT mice were perfused in situ, via the hepatic vein, with cold phosphate-buffered saline to remove excess haemoglobin. Livers were flash frozen in liquid nitrogen and stored at −80 °C. Samples were prepared, and differential protein expression between *Fmo5*^*−/−*^ and WT liver samples was quantified by fluorescence two-dimensional difference gel electrophoresis (2D-DIGE), followed by image analysis, essentially as described [31]. The criteria for identifying differentially expressed proteins were >1.5-fold difference in spot intensity and a statistical *t*-test *P* < 0.01. Selected protein spots were picked using an Ettan Spot Picker system (GE Life Sciences). Proteins were digested with trypsin and identified by liquid chromatography tandem mass spectrometry (MS), essentially as described [32]. Raw MS data were searched against the IPI mouse database (20100214; 56,737 sequences) using the Mascot search engine. Identifications were accepted when two or more usable unique peptide sequences were matched to a protein, using a significance threshold of >0.05 and an ion score cut off of >20.

### 2.10 Western blot analysis

Liver tissue was homogenized in ice-cold 1% Triton X-100, 140 mM NaCl, 10 mM Tris pH8, 1 mM EDTA, 1 mM PMSF containing Halt™ Protease and Phosphatase Inhibitor Cocktail (Thermo Fisher Scientific, Loughborough, Leicestershire, UK) at 25 Hz for 30 s in a Tissue Lyser II (Qiagen, Crawley, Surrey, UK). Samples were left to cool on ice for 1 min and homogenized for a further 30 s at 25 Hz. Homogenates were rotated on a spinning wheel for 15 min at 4 °C, then centrifuged at 12,000 × *g* for 20 min at 4 °C. Supernatants were analyzed by SDS-PAGE and western blotting. For FMO5, the blot was incubated with Rabbit anti-FMO5 polyclonal Antibody, 13699-1-AP (Proteintech, Chicago, IL) and then with a horse radish peroxidase-conjugated secondary antibody (Donkey Anti-Rabbit IgG H&L (HRP)), ab97064 (Abcam, Cambridge, MA). Blots were developed using enhanced chemiluminescence (ECL), as described [33], using 4-iodophenylboronic acid (Thermo Fisher Scientific) as an enhancer. Signal was detected using an LAS-1000 image reader (Fujifilm UK, Ltd., Bedford, Beds, UK) with software version 2.6. For malic enzyme 1 (ME1) liver tissue was homogenized at 4 °C in RIPA lysis buffer (Sigma–Aldrich) containing Halt™ Protease and Phosphatase Inhibitor Cocktail. Homogenates were agitated continuously for 2 h at 4 °C, then centrifuged at 12,000 × *g* for 20 min at 4 °C. Supernatants were analyzed by SDS-PAGE and western blotting. The blot was incubated with Anti-ME1 antibody, ab84561 (Abcam). The secondary antibody was Alexa Fluor 800 goat anti-rabbit IgG (Life Technologies, Paisley, Scotland, UK). For loading control the blot was incubated with a monoclonal anti-mouse beta-actin (Sigma–Aldrich) and an anti-mouse Alexa Fluor 680 secondary antibody (Life Technologies), then imaged using an Odyssey system (LI-COR Biosciences Ltd., Cambridge, Cambs, UK).

### 2.11 Quantitative real-time (qRT) PCR

Liver RNA was isolated using Tri Reagent (Sigma–Aldrich) and cDNA synthesized using a Precision QScript Reverse Transcriptase kit (Primer Design Ltd, Southampton, Hants, UK). qRT PCR was performed as described previously [25] and mRNAs were quantified by the ΔΔC_T_ method [34]. Primer sequences forward (F) and reverse (R) were: Srebp2F 5′ TGAAGGACTTAGTCATGGGGAC 3′ and Srebp2R 5′ CGCAGCTTGTGATTGACCT 3′, Hmgcs1F 5′ CCTGGACCGCTGCTATTCT 3′ and Hmgcs1R 5′ CAGTTTACCAATATGGTGAGTGAAAGA 3′, HmgcrF 5′ CCGAATTGTATGTGGCACTGT 3′ and HmgcrR 5′ TTATCTTTGATCTGTTGTGAACCAT 3′, SSF 5′ ATGGAGTTCGTCAAGTGTCTAGG 3′ and SSR 5′ GCTGCCGTATGTCCCCATC 3′, Cyp7a1F 5′ ACACATACCAATAAGAAGAGCATTT 3′ and Cyp7a1R 5′ GACCAGAATAACCTCAGACTCATA 3′, Cyp27a1F 5′ GATGAGACAGGAGGGCAAGTA 3′ and Cyp27a1R 5′ TGCGATGAAGATCCCATAGGT 3′, SRB1F 5′ TTCTGGGGTCTTCACTGTCTT 3′ and SRB1R 5′ TCTTGCTGAGTCCGTTCCAT 3′, Abcg5F 5′ GTCATCGCCACGGTCATTT 3′ and Abcg5R 5′ AGAGCAGCAGAGAAATATCCAAA 3′, Abcg8F 5′ GCAGATTCAATTTAATGGACACCTT 3′ and Abcg8R, 5′ CATAGAGTGGATGCGAGTTCAG 3′, Abcb11F 5′ TGGTCAATTCCTTCACTAACATCT 3′ and Abcb11R 5′ AAGCGAATCCTGTCAGCATTT 3′, ME1F 5′ GGCCTGCGGACTGAGACACATCGA 3′ and ME1R 5′ AAACAGTGGCCATCTTTTCTTTGTA 3′.

A geNorm™ kit and geNorm software (Primer Design Ltd.) were used to determine the most suitable housekeeping gene for use as internal reference.

### 2.12 Squalene synthase assay

Microsomal membrane vesicles were isolated from liver and squalene synthase activity was measured using farnesyl pyrophosphate-[1-^3^H(N)], triammonium salt (Perkin Elmer, Seer Green, Bucks, UK), as described previously [35], except that the incubation time was 45 min. Radioactive squalene was resolved by thin-layer chromatography, using 5% toluene in hexane, and revealed by incubating the plate with iodine pearls. The spot was marked and the plate left overnight to release iodine. The spot was then scraped into scintillation fluid and radioactive squalene measured by liquid scintillation spectrometry. Protein was assayed using the DC protein assay (Bio-Rad Laboratories Ltd., Hemel Hempstead, Herts, UK).

### 2.13 Quantification of acetyl-CoA

For total hepatic acetyl-CoA, frozen liver (0.1 g) was homogenized on ice in 0.5 ml of ice-cold 5% (w/v) perchloric acid (PCA) using an UltraTurrax tissue disintegrator. For cytosolic acetyl-CoA, liver was homogenized in ice-cold 225 mM mannitol, 75 mM sucrose, 0.1 mM EDTA, 5 mM MOPS pH7.4 using a Potter Elvehjem homogenizer. The homogenate was centrifuged at 700 × *g* for 10 min and the supernatant was centrifuged at 12,000 × *g* for 10 min to pellet mitochondria. The postmitochondrial supernatant was centrifuged at 100,000 × *g* for 90 min. All centrifugation steps were performed at 4 °C. The resulting supernatant was acidified by adding PCA to a final concentration of 5%. Acetyl-CoA was measured as described [36].

### 2.14 Statistical analysis

Statistical analyses were performed using an unpaired, two-tailed *t*-test or one-way ANOVA, as appropriate. Significance level *P* < 0.05.

## 3 Results

### 3.1 *Fmo5*^*−/−*^ mice exhibit an age-related lean phenotype, with reduced gains in body weight and fat deposits

**Fig. 2:**
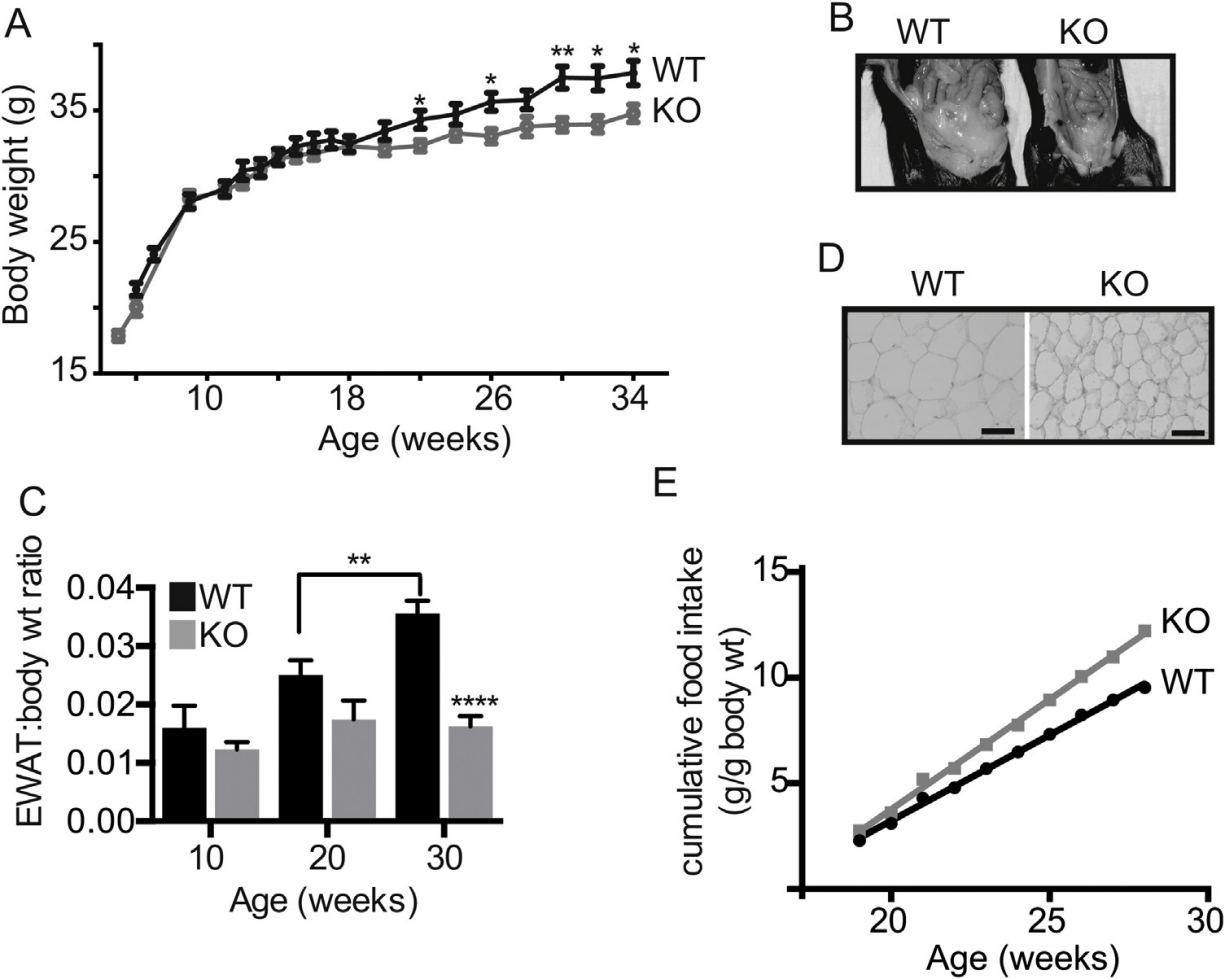
*Fmo5*^−/−^ mice exhibit a lean phenotype. (A) Body weight plotted as means ± SEM (WT, *n* = 8; KO, *n* = 7), *P < 0.05, **P < 0.01. (B) Internal abdominal view of 30-week-old WT and *Fmo5*^−/−^ (KO) mice. (C) Ratio of weight of EWAT to body weight of WT and *Fmo5*^−/−^ (KO) mice. 10 week: *n* = 4 (WT), 3 (KO); 20 week: *n* = 10 (WT), 5 (KO); 30 week: *n* = 11 (WT), 8 (KO). Data are expressed as means ± SEM. **P < 0.01; ****P < 0.0001. (D) Sections of epididymal WAT of 30-week-old WT and *Fmo5*^−/−^ (KO) mice stained with haematoxylin and eosin. Scale bar = 100 μm. (E) Food intake expressed as cumulative food intake in grams per g of body weight of WT and *Fmo5*^−/−^ (KO) mice. *n* = 8 (WT), 7 (KO).

*Fmo5*^*−/−*^ mice appeared healthy and bred normally. From birth to about 20 weeks of age the weight of male KO and WT mice fed a standard chow diet was similar (Fig. 2A). From 20 weeks of age both KO and WT mice continued to gain weight. However, from this age the rate of weight gain of the KO mice was less than that of WT mice (Fig. 2A), and by 30 weeks the weight of KO mice (33.92 ± 0.53 g, *n* = 7) was 10% less than that of WT animals (37.50 ± 0.85 g, *n* = 8) (*P* < 0.01). The difference in weight increased with age and by 52 weeks KO mice (35.46 ± 0.50 g, *n* = 5) weighed 17% less than WT mice (42.96 ± 0.66 g, *n* = 5) (*P* < 0.001). Female mice displayed a similar age-related difference in weight gain (data not shown) and at 30 weeks of age the weight of female KO mice (23.79 ± 0.71 g, *n* = 10) was 14% less than that of WT mice (27.75 ± 0.81 g, *n* = 6) (*P* < 0.01). As age-related differences in weight gain between KO and WT mice were similar in both male and female mice, subsequent analysis was done on male animals unless stated otherwise.

At 30 weeks of age, the KO mice were leaner than WT mice, had less subcutaneous and inguinal fat and less fat surrounding internal organs (Fig. 2B). The epididymal fat pads of KO mice (0.47 ± 0.05 g, *n* = 8) weighed 63% less than those of WT mice (1.27 ± 0.11 g, *n* = 11) (*P* < 0.0001). At 10 weeks of age the ratio of the weight of epididymal white adipose tissue (EWAT) to body weight of WT and KO mice was similar (Fig. 2C). In WT mice it increased significantly with age (*P* < 0.01), reaching 0.036 ± 0.002 (*n* = 11) by 30 weeks (Fig. 2C). In contrast, in KO mice the ratio was relatively constant with age and at 30 weeks was 0.016 ± 0.002 (*n* = 8), 56% less than that of WT mice (*P* < 0.0001). Inactivation of the *Fmo5* gene therefore leads to an age-related reduction in the rate of increase in body weight, which is accompanied by a lack of increase in fat depot size as the KO mice age.

At 30 weeks of age the diameter of epididymal adipocytes from the KO mice (57.22 ± 1.60 μm, *n* = 3) was less than that of those from WT mice (78.80 ± 2.88 μm, *n* = 3) (*P* < 0.01) (Fig. 2D), equivalent to a decrease in cell volume of 62%. This corresponds closely to the 63% decrease in the weight of epididymal fat pads of the KO mice, indicating that the difference in weight of epididymal fat between KO and WT mice was due to a difference in adipocyte volume.

Over a 12-week period from 16 weeks of age, the cumulative intake of food by KO mice was ∼28% more than by WT mice, when measured per g of body weight (Fig. 2E). The increased intake of food by the KO mice was evident from 20 weeks of age, which coincides with the time at which their rate of weight gain begins to slow in comparison with WT mice (Fig. 2A). At 30 weeks of age, although the FMO5 KO mice weighed 10% less than WT animals, their food intake per animal per day was 14% more than that of WT mice (data not shown). Therefore, the reduction in the rate of weight gain and in the amount of WAT in the KO mice occurred despite an increase in food intake.

### 3.2 *Fmo5*^*−/−*^ mice have no impairment of lipid import into or export from adipocytes

There were no significant differences between 30-week-old KO and WT mice in the plasma concentrations of triglycerides (KO, 0.83 ± 0.03 mmol/l, *n* = 7; WT, 0.89 ± 0.07 mmol/l, *n* = 10), non-esterified fatty acids (NEFA) (KO, 0.88 ± 0.09 mmol/l, *n* = 4; WT, 0.74 ± 0.07 mmol/l, *n* = 4) or glycerol (KO, 0.35 ± 0.02 mmol/l, *n* = 4; WT, 0.38 ± 0.01 mmol/l, *n* = 4), indicating that the reduced accumulation in *Fmo5*^*−/−*^ mice of adipose tissue triacylglycerol was not the result of impaired lipid import into or export from adipocytes. The plasma concentration of NEFA after a 16-h fast increased to 1.40 ± 0.10 mmol/l (*n* = 4) in KO mice and to 1.36 ± 0.13 mmol/l (*n* = 4) in WT mice. Thus, despite having less stored fat, *Fmo5*^*−/*−^ mice were able to mobilize triglycerides from WAT normally in response to an overnight fast.

### 3.3 *Fmo5*^*−/−*^ mice do not increase ectopic fat stores

Histological analysis revealed that there was no difference in the amount of lipid present in the livers of KO and WT mice (data not shown). This indicates that the decreased accumulation of triglycerides in WAT of *Fmo5*^*−/−*^ mice was not a consequence of an increase in ectopic fat storage and suggests that export of triglycerides from liver is not impaired in these animals.

### 3.4 *Fmo5*^*−/−*^ mice have reduced plasma glucose and cholesterol

**Fig. 3:**
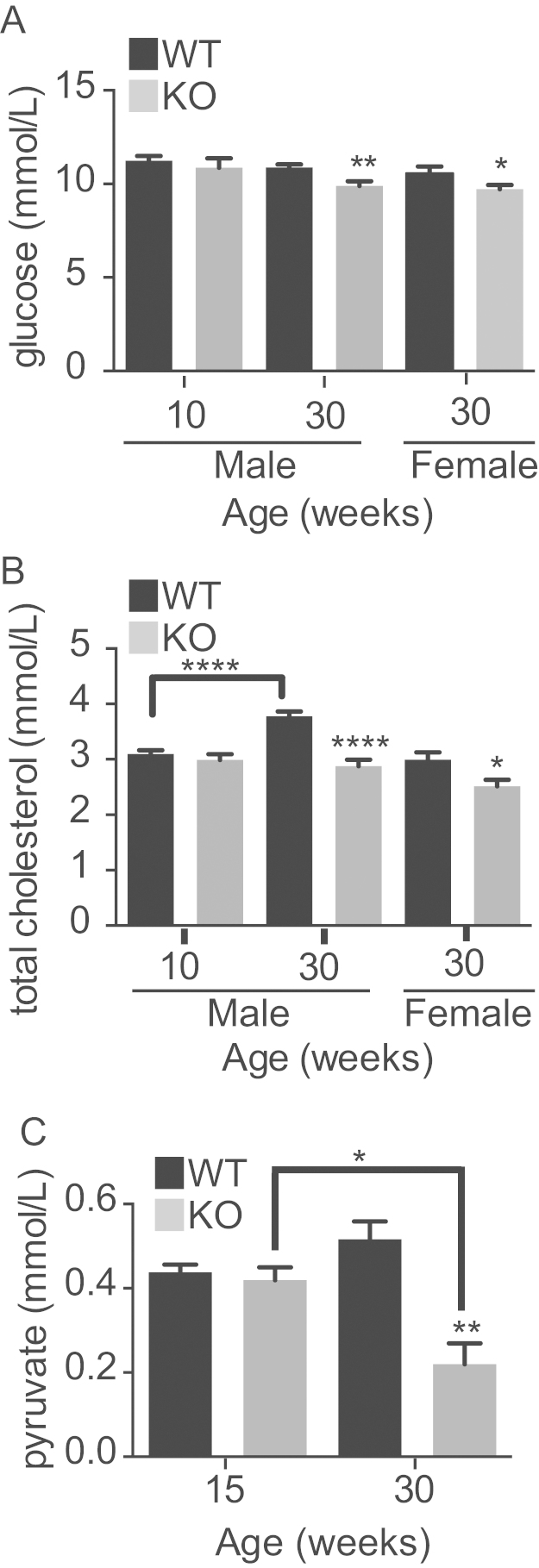
*Fmo5^−/−^* mice have lower plasma concentrations of glucose, cholesterol and pyruvate. (A) Plasma concentration of glucose in 10- and 30-week-old male and 30-week-old female WT and *Fmo5*^−/−^ (KO) mice. 10 week: *n* = 6 (WT), 3 (KO); 30-week male: *n* = 19 (WT), 11 (KO); 30-week female: *n* = 9 (WT), 13 (KO). (B) Plasma concentration of total cholesterol in 10- and 30-week-old male and 30-week-old female WT and *Fmo5*^−/−^ (KO) mice. 10 week: *n* = 11 (WT), 8 (KO); 30-week male: *n* = 23 (WT), 18 (KO); 30-week female: *n* = 6 (WT), 10 (KO). (C) Plasma concentration of pyruvate in 15- and 30-week-old male WT and *Fmo5* (KO) mice, *n* = 4 (15 week), 5 (30 week). Data are expressed as means ± SEM. *P < 0.05, **P < 0.01, ****P < 0.0001.

The plasma concentration of glucose in 10-week-old male *Fmo5*^*−/−*^ and WT mice was similar (Fig. 3A). At 30 weeks of age it was unchanged in WT mice (10.92 ± 0.23 mmol/l, *n* = 19), but was lower in KO mice (9.07 ± 0.63 mmol/l, *n* = 11) (*P* < 0.05). Thirty-week-old female KO mice also had lower plasma glucose than their WT counterparts (Fig. 3A). At 30 weeks, both KO and WT male mice responded to a 12-h overnight withdrawal of food by decreasing their plasma glucose. Again, the plasma concentration of glucose in KO mice (6.60 ± 0.09 mmol/l, *n* = 5) was significantly lower than in WT mice (7.90 ± 0.35 mmol/l, *n* = 8) (*P* < 0.01). The urinary concentration of glucose of KO (1.69 ± 0.15 mmol/l, *n* = 3) and WT mice (2.12 ± 0.51 mmol/l, *n* = 3) was similar, indicating that the reduced plasma glucose of KO mice was not a consequence of enhanced urinary excretion of glucose.

There was no difference in liver glycogen content of KO (66.83 ± 4.88 μg/g liver, *n* = 5) and WT (63.84 ± 5.16 μg/g liver, *n* = 6) mice. Thus, the reduction in plasma glucose in the *Fmo5*^*−/−*^ mice is not due to increased storage of glucose in the form of glycogen in the liver or to decreased mobilization of glucose from liver glycogen.

The plasma concentration of total cholesterol in 10-week-old male KO and WT mice was similar (Fig. 3B). At 30 weeks of age the concentration in WT mice had increased, but in KO animals remained the same, 24% less than that in WT animals (Fig. 3B). In 30-week-old mice, plasma cholesterol in female WT animals was lower than in male WT animals and similar to that in male KO mice (Fig. 3B). As was the case for male mice, in 30-week-old female mice plasma cholesterol was lower in KO than in WT animals (Fig. 3B). In 30-week-old male animals plasma concentrations of both HDL (KO, 1.61 ± 0.21 mmol/l, *n* = 7; WT, 2.43 ± 0.10 mmol/l, *n* = 10) (*P* < 0.01) and LDL (KO, 0.36 ± 0.02 mmol/l, *n* = 7; WT, 0.48 ± 0.02 mmol/l, *n* = 10) (*P* < 0.001) were lower, by 34% and 25% respectively, in the KO mice. There was no significant difference between KO and WT mice in the ratio of total cholesterol to HDL.

### 3.5 *Fmo5*^*−/−*^ mice have enhanced energy expenditure

**Fig. 4:**
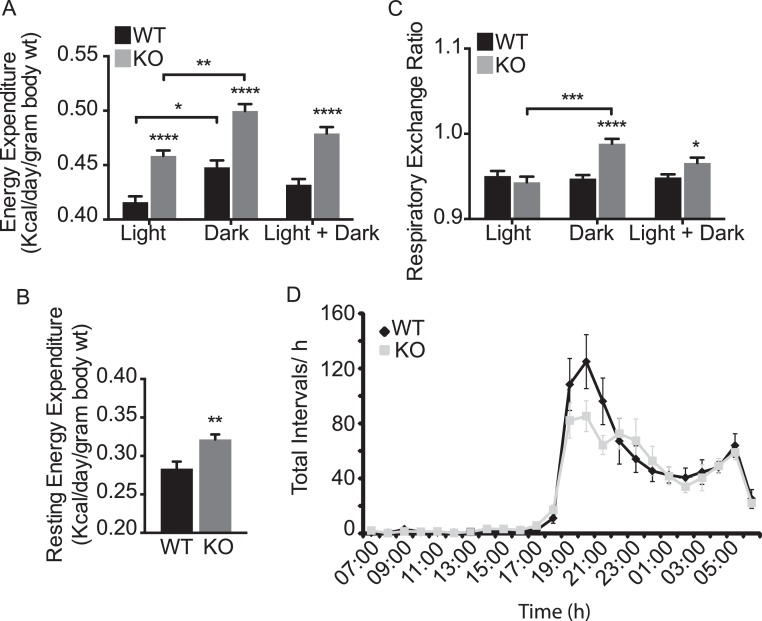
*Fmo5^−/−^* mice have enhanced energy expenditure, but no increase in voluntary exercise. (A) Energy expenditure in the light, dark and combined light and dark phases. (B) REE. (C) RER for light, dark and combined light and dark phases. Light phase (L, 07:00 to 19:00), dark phase (D, 19:00 to 07:00). Parameters were measured over a 72-h period for 30-week-old male WT (*n* = 6) and *Fmo5*^−/−^ (KO) (*n* = 6) mice. Values are means ± SEM. *P < 0.05, **P < 0.01, ***P < 0.001, ****P< 0.0001. (D) Voluntary wheel running of 30-week-old male WT and *Fmo5*^−/−^ (KO) mice was assessed over seven days. Actogram shows average interval count/h ± SEM for a single day.

Total energy expenditure, as measured by oxygen consumption, was significantly higher in *Fmo5*^*−/−*^ mice than in WT animals in both the light and dark phases (Fig. 4A), which suggests that oxidation of fuel substrates is continuously higher in the KO mice. Resting energy expenditure (REE), a measure of basal metabolic rate plus energy expended in processing food, also is higher in KO than in WT mice (Fig. 4B). More than 80% of the enhanced whole-body energy expenditure of *Fmo5*^*−/−*^ mice is thus attributable to an increase in REE.

The respiratory exchange ratio (RER) reflects the relative contributions of carbohydrate and fat oxidation to total energy expenditure. During the light phase the RER for FMO5 KO mice (0.943 ± 0.007) was similar to that for WT mice (0.950 ± 0.006) (Fig. 4C). During the dark phase the RER of WT animals remained the same as that for the light phase. In contrast, in the dark phase the RER of the KO mice increased to 0.989 ± 0.006 (Fig. 4C). The results indicate that during the light phase the proportion of carbohydrate and fat oxidized by KO and WT mice is similar and for WT mice remains the same during the dark phase. However, during the dark phase the KO mice increase the proportion of carbohydrate oxidized. Urinary concentrations of urea and creatinine were similar in KO and WT mice (data not shown), indicating that the KO mice did not increase protein breakdown to maintain energy balance.

### 3.6 *Fmo5*^*−/−*^ mice do not exhibit increased physical activity

The physical activity of *Fmo5*^*−/−*^ and WT mice, as assessed by voluntary wheel running over a period of seven days, was similar, with a normal increase in nocturnal activity for both sets of mice (Fig. 4D). There was no significant difference between the KO and WT mice in either the distance run or the speed of running (data not shown). Therefore the enhanced whole-body energy expenditure of *Fmo5*^*−/−*^ mice was not a consequence of increased physical activity.

### 3.7 In *Fmo5*^*−/−*^ mice fatty acid oxidation is increased in EWAT and decreased in skeletal muscle

At 20 weeks of age, the time at which KO mice begin to show a reduction in weight gain, the rate of oxidation of [^14^C]palmitate in EWAT of KO animals (219 ± 30 μmol palmitate oxidized/fat pad/h, *n* = 4) was 55% more than that of WT animals (141 ± 17 μmol palmitate oxidized/fat pad/h, *n* = 10) (*P* < 0.05). The increased rate of fatty acid oxidation would contribute to depletion of lipid stores in EWAT of KO mice.

Heart and skeletal muscle are tissues that oxidize lipid as their major energy source. In 20-week-old mice the rate of [^14^C]palmitate oxidation in cardiac muscle was similar in KO and WT mice (data not shown). However, in resting soleus muscle it was 32% lower in KO (1255 ± 74 μmol palmitate oxidized/g/h, *n* = 5) than in WT animals (1843 ± 224 μmol palmitate oxidized/g/h, *n* = 4) (*P* < 0.05).

### 3.8 Proteins involved in carbohydrate metabolism or in cholesterol or lipid synthesis are down regulated in *Fmo5*^*−/−*^ mouse liver

**Fig. 5:**
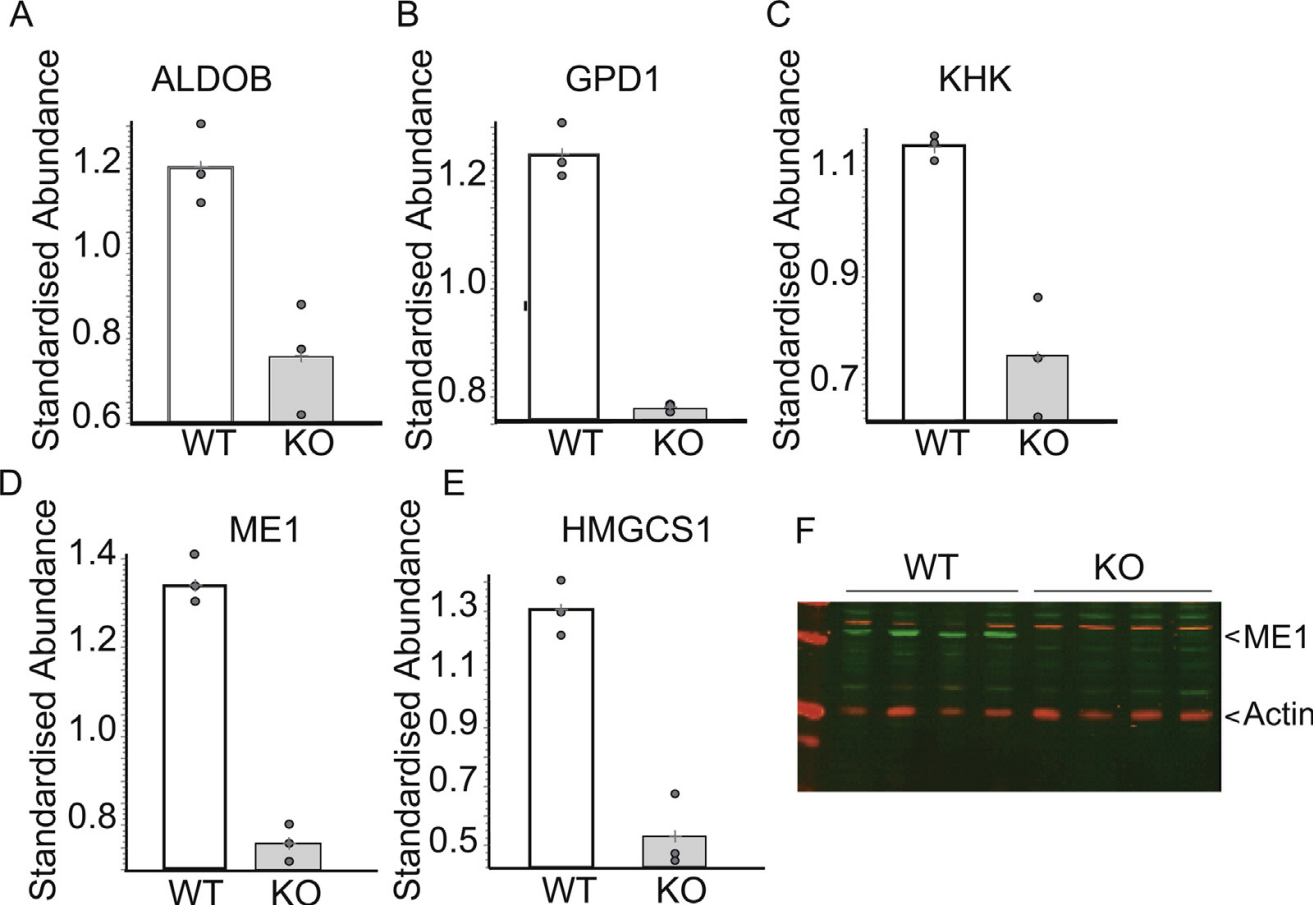
Five key metabolic enzymes are down regulated in the liver of *Fmo5*^−*/*−^ mice. Relative abundance of aldolase B (ALDOB) (A), glycerol 3-phosphate dehydrogenase (GPD1) (B), ketohexokinase (KHK) (C), malic enzyme 1 (ME1) (D) and HMG-CoA synthase 1 (HMGCS1) (E) in liver of WT and *Fmo5*^−/−^ (KO) mice determined via proteomic analysis. (F) Western blot analysis of liver lysates of WT and *Fmo5*^−/−^ (KO) mice. The blot was incubated with antibodies against ME1 and actin, as a loading control, and developed as described in Section 2.

The liver plays an important role in metabolic homeostasis and is an organ in which the *Fmo5* gene is highly expressed [10]. To gain an insight into the basis of the metabolic phenotype of the *Fmo5*^*−/−*^ mice we performed a proteomic analysis of liver to identify proteins that differed in abundance between KO and WT mice and, thus, might contribute to the phenotype. Five proteins were down regulated in KO animals. All are enzymes involved in carbohydrate metabolism or in cholesterol or lipid biosynthesis (Fig. 5): aldolase B and ketohexokinase, involved in glucose and fructose metabolism; glycerol 3-phosphate dehydrogenase (GPD1), a cytosolic protein important for the production of NAD^+^ and for transporting reducing equivalents from the cytosol to mitochondria; β-hydroxy-β-methylglutaryl-CoA (HMG-CoA) synthase 1, a cytosolic protein involved in cholesterol biosynthesis; and cytosolic malic enzyme (ME1), a lipogenic enzyme that catalyzes the oxidative decarboxylation of malate to pyruvate, in the process producing NADPH for use in lipid and cholesterol biosynthesis. Down-regulation of aldolase B, a glycolytic enzyme, and ME1 would be expected to result in a decrease in the production of pyruvate. This is supported by the finding that the plasma concentration of pyruvate in 30-week-old animals is 57% lower in *Fmo5*^*−/−*^ mice than in WT animals (Fig. 3C). The difference in plasma pyruvate is age related, as in 15-week-old animals the concentration is the same in KO and WT mice (Fig. 3C).

The lower abundance of HMG-CoA synthase 1 in the liver of KO mice was not the result of a difference in the abundance of the corresponding mRNA (Table 1). In contrast, the mRNAs for two other enzymes involved in cholesterol biosynthesis, HMG-CoA reductase and squalene synthase, were more abundant in the livers of KO than of WT mice, as was the mRNA for sterol regulatory element-binding protein-2 (SREBP-2), a transcription factor that up-regulates the transcription of genes encoding several enzymes involved in cholesterol synthesis (Table 1). Although the abundance of the mRNA for squalene synthase was greater in the livers of KO than of WT mice, neither the amount nor activity of the encoded protein differed significantly between KO and WT mice (data not shown). The abundance in liver of mRNAs for proteins involved in cholesterol uptake and transport or in bile acid synthesis did not differ between KO and WT mice (Table 1).

All the carbon in cholesterol is derived from acetate, via acetyl-CoA. The amounts of total and cytosolic acetyl-CoA in liver were not significantly different between KO and WT mice (data not shown).

Western blotting revealed that, in contrast to WT mice, in *Fmo5*^*−/−*^ mice ME1 was not detectable in the liver (Fig. 5F). However, as was the case for HMG-CoA synthase 1 (see above), the abundance of the corresponding ME1 mRNA in the livers of KO and WT mice was not significantly different. Therefore down regulation of the expression of HMG-CoA synthase 1 and ME1 in *Fmo5*^*−/−*^ mice is mediated at the translational or post-translational level.

## 4 Discussion

Mice in which the *Fmo5* gene had been inactivated appeared healthy but exhibited a lean phenotype, which was age-related, becoming apparent only at about 20 weeks of age. From this age WT mice continued to increase body weight and this was accompanied by increases in the amount of lipid stored in WAT and in the plasma concentration of cholesterol. In contrast, as they aged *Fmo5*^*−/−*^ mice, despite eating more than their WT counterparts, were resistant to weight gain and their fat deposits and plasma cholesterol remained the same as that of 10-week-old mice. In addition, at 30 weeks of age the plasma concentration of glucose was lower in KO than in WT mice. The main characteristics of the phenotype, reduced weight gain and lower plasma concentrations of glucose and cholesterol, were evident also in female *Fmo5*^*−/−*^ mice and, thus, the phenotype is gender-independent.

*Fmo5*^*−/−*^ mice suffered no loss of appetite or decrease in physical activity, indicating that the phenotype is unlikely to be a consequence of illness or general malaise. Indeed, disruption of the gene encoding FMO5 resulted in a relatively ‘healthy’ phenotype, with mice being protected against a number of age-related metabolic changes that could adversely affect wellbeing. The increased food consumption of the *Fmo5*^*−/−*^ mice was likely a response to the lower amounts of fat reserves of these animals.

The lean phenotype of *Fmo5*^*−/−*^ mice was associated with enhanced whole-body energy expenditure, most of which was due to higher REE, with no increase in physical activity. The basis of the enhanced REE is unclear.

The increased rate of fatty acid oxidation in EWAT of KO mice at 20 weeks of age would contribute to the depletion of triglyceride stores in this tissue and, hence, to reduction of adipocyte volume. At the same age, the lower rate of fatty acid oxidation in skeletal muscle of KO than of WT mice suggests a switch to increased use of carbohydrate as fuel in this tissue in KO mice. This is supported by the RER of 30-week-old mice, which indicates that during the dark phase, the period in which the mice are most active, the ratio of carbohydrate to fat utilized as fuel was higher in KO than in WT mice. The increased use by KO mice of carbohydrate as fuel is possibly a response to the diminished stores of lipid in WAT of these animals and might also contribute to their lower plasma concentration of glucose. In WT mice the *Fmo5* gene is not expressed in WAT or skeletal muscle (data not shown), indicating that the effects in these tissues of disruption of *Fmo5* are indirect.

Three of the proteins down regulated in the liver of *Fmo5*^*−/−*^ mice, aldolase B, ketohexokinase and GPD1, are involved in carbohydrate metabolism. Aldolase B is a glycolytic enzyme that catalyzes the production of glyceraldehyde 3-phosphate and dihydroxyacetone phosphate (DHAP), from fructose 1,6-bisphosphate. Ketohexokinase (or fructokinase) catalyzes the conversion of fructose to fructose 1-phosphate, which can be converted to glyceraldehyde and DHAP in a reaction catalyzed by aldolase B. Both glyceraldehyde and DHAP can be converted to the glycolytic intermediate glyceraldehyde 3-phosphate, via reactions catalyzed by triose kinase and triose phosphate isomerase respectively. For glycolysis to proceed glyceraldehyde 3-phosphate must be converted to 1,3-bisphosphoglycerate, in a reaction which requires NAD^+^ and produces NADH. GPD1 catalyzes the oxidation of cytosolic NADH by DHAP to produce NAD^+^, which re-enters glycolysis, and glycerol 3-phosphate, which can be combined with fatty acids to form triglycerides. The expected consequence of the down regulation of aldolase B, ketohexokinase and GPD1 in the liver of *Fmo5*^*−/−*^ mice is slowing of the entry of fructose into glycolysis and of the metabolism of glucose via glycolysis and, thus, a decrease the amount of pyruvate produced by this pathway. This is supported by the finding that the plasma concentration of pyruvate was lower in KO than in WT mice. In addition, down regulation of GPD1 would be expected to moderate the biosynthesis of triglycerides, via its effect on production of glycerol 3-phosphate, providing a potential explanation for the reduced fat deposits of these animals.

Aldolase B can catalyze the reverse reaction, producing fructose 1,6-bisphosphate from DHAP and glyceraldehyde 3-phosphate, a key step in gluconeogenesis. Down regulation of aldolase B would, therefore, be expected to slow both glycolysis and gluconeogenesis, suggesting that *Fmo5*^*−/−*^ mice do not increase gluconeogenesis in response to a lower plasma concentration of glucose. The similarity in liver glycogen content of the KO and WT mice indicates that the former do not respond to lower plasma glucose by increasing the mobilization of glucose from glycogen.

The lower plasma concentration of cholesterol in *Fmo5*^*−/−*^ mice was accompanied by lower abundance in liver of HMG-CoA synthase 1, which catalyzes the first committed step in isoprenoid biosynthesis. It has been shown that modulation of HMG-CoA synthase 1 activity influences cholesterol biosynthesis [37,38] and the enzyme is negatively regulated, via a feedback mechanism, in response to cholesterol feeding [39]. In contrast, in *Fmo5*^*−/−*^ mice down regulation of the amount of HMG-CoA synthase 1 occurred in the context of lower, not higher, plasma cholesterol. However, the induction in *Fmo5*^*−/−*^ mice of mRNAs encoding HMG-CoA reductase, which catalyzes the rate-limiting step in cholesterol biosynthesis, squalene synthase, which catalyzes the first step in the pathway that produces cholesterol from farnesyl pyrophosphate, and the transcription factor SREBP-2 is a normal response to low plasma cholesterol [40,41]. But, despite this, the concentration of plasma cholesterol in the KO mice remained low. In *Fmo5*^*−/−*^ mice down regulation of HMG-CoA synthase 1 protein occurred in the absence of a change in the amount of its mRNA, indicating that, in these animals, regulation of expression of HMG-CoA synthase 1 was independent of transcription and also of that of other key enzymes in the pathway.

Acetyl-CoA is a product of fatty acid and glucose catabolism. Depending on the needs of a cell or organism, acetyl-CoA can be used for energy production, by entering the citric acid cycle, or for the biosynthesis of fatty acids, ketone bodies or cholesterol. The concentration of cytosolic acetyl-CoA in the livers of KO and WT mice was similar, suggesting that the impact on fat and cholesterol production in the KO mice was primarily due to down regulation of key synthetic enzymes, not to a shortage of the primary building blocks.

The lower plasma concentration of pyruvate in the *Fmo5*^*−/−*^ mice could be explained by the down regulation in liver of the glycolytic enzyme aldolase B (see above) and of ME1, which catalyzes the production of pyruvate from malate. It has been shown, in cell culture, that glucose, through the glycolytic product pyruvate, induces the amount and activity of ME1 [42]. Thus, in *Fmo5*^*−/−*^ mice reduction in the production of pyruvate, as a consequence of down regulation of aldolase B, might contribute to the down regulation of ME1.

Expression of ME1 is subject to regulation at several stages: transcription, stabilization of nuclear RNA and degradation of cytoplasmic RNA; and it is regulated independently in response to a high-carbohydrate diet and by thyroid hormone [43]. In *Fmo5*^*−/−*^ mice the abundance of ME1 is apparently regulated by a different mechanism: either via a block in the translation of the mRNA or an increase in the rate of degradation of the protein.

The reaction catalyzed by ME1 is an important source of NADPH for use in anabolic reactions, including the biosynthesis of fatty acids and cholesterol. Repression of ME1 decreases the cellular concentration of NADPH [42,44]. Reduction in the concentration of NADPH, as a consequence of down regulation of ME1, would be expected to moderate the biosynthesis of both fatty acids and cholesterol, thus providing a potential explanation for the reduced fat deposits and lower plasma cholesterol that are characteristic of the metabolic phenotype of *Fmo5*^*−/−*^ mice.

In liver biopsies of type-2 diabetics, *FMO5* was one of 134 genes found to be repressed [45]. The phenotype of the *Fmo5*^*−/−*^ mice reveals that in the absence of FMO5 weight and plasma glucose are reduced. This suggests strongly that the reduction in the expression of *FMO5* in type-2 diabetics is a response to this aberrant metabolic condition in an attempt to restore homeostasis.

Another FMO KO mouse line, one that lacks genes encoding FMO1, FMO2 and FMO4, also exhibits a lean phenotype, with reduced body weight and fat deposits [25]. However, there are distinct differences between this KO mouse line and *Fmo5*^*−/−*^ mice. The phenotype of mice lacking *Fmo1*, *Fmo2* and *Fmo4* genes, which is attributed to lack of FMO1, is evident from as early as six weeks of age and is characterized by enhanced whole-body energy expenditure and REE with no change in RER (indicating an increase in both fat and carbohydrate oxidation), increased capacity for exercise, elevated fatty acid oxidation in skeletal muscle, but not in WAT, higher plasma glucose and evidence for the operation of a futile fuel cycle in WAT [25]. In contrast, the phenotype of *Fmo5*^*−/−*^ mice is age-related, becoming apparent only after 20 weeks of age, and is characterized by lower plasma concentrations of glucose and cholesterol, fatty acid oxidation that is reduced in skeletal muscle but elevated in WAT, and no increase in physical activity. In common with mice that lack FMO1, FMO2 and FMO4, mice that lack FMO5 exhibit enhanced whole-body energy expenditure and REE, but have increased RER, indicating a switch from fat to carbohydrate oxidation. Thus, both FMO5 and FMO1 are metabolic regulators. However, whereas FMO1 acts as a regulator of energy homeostasis [25], our results indicate that FMO5 regulates metabolic ageing via pleiotropic effects. These include positive modulation of cholesterol biosynthesis, via promoting the expression in liver of enzymes involved in the cholesterol biosynthetic pathway and the production of NADPH, and also promoting the hepatic expression of enzymes involved in glycolysis/gluconeogenesis and in the formation of glycerol 3-phosphate for the biosynthesis of triglycerides.

FMO5 is classified as an oxidoreductase and it is possible that its effects are mediated via modulation of the cellular redox state. However, in vivo substrates of FMO5 have yet to be identified and the mechanism by which the enzyme exerts its pleiotropic effects remains to be established.

Our results have potential implications for humans, indicating that interindividual variation in *FMO5* expression [20–22] may contribute to differences in fat deposits and plasma cholesterol and that induction of *FMO5* expression by some therapeutics [19] may have adverse effects on the metabolic health of patients.

## Figures and Tables

**Table 1:** Relative difference in the abundance of mRNAs in the liver of WT and *Fmo5*^−*/*−^ mice.

mRNA	Relative expression, KO to WT
*Cholesterol biosynthesis*	
HMG CoA synthase (HMGCS1)	1.71 ± 0.47
HMG CoA reductase (HMGR)	2.41 ± 0.46*
Squalene synthase (SS)	3.23 ± 0.57*
Sterol responsive element binding protein (SREBP-2)	2.45 ± 0.28*

*Cholesterol uptake and transport*	
Scavenger receptor (SR-B1)	1.24 ± 0.21
Abcg 5	1.74 ± 0.26
Abcg 8	1.60 ± 0.25

*Bile acid synthesis*	
Cholesterol 7a hydroxylase (CYP7a1)	1.22 ± 0.49
Cholesterol 27a hydroxylase (CYP27a1)	0.78 ± 0.20

The abundance of each mRNA was determined by qRT-PCR. Data are presented as relative abundance in *Fmo5*^*−/−*^ (KO) compared to WT mice. Values are expressed as means ± S.E.M, *n* = 6–9.

* *P* < 0.05.
